# Miniaturized flexible skin moisture sensor with optimized coil for enhanced wireless power efficiency

**DOI:** 10.1038/s41598-026-38764-5

**Published:** 2026-02-10

**Authors:** Hyejun Kim, Seongu Kim, Changyu Yeo, Minkyung Kim, Weonho Shin, Jeonghyun Kim

**Affiliations:** 1 Department of Electronic Convergence Engineering, Kwangwoon University, Seoul, 01897 South Korea; 2 Department of Electronic Materials Engineering, Kwangwoon University, Seoul, 01897 South Korea

**Keywords:** Energy science and technology, Engineering

## Abstract

Efficient wireless power transfer is essential for the stable operation of battery-free wearable sensors. Especially for Near Field Communication (NFC)-based sensors, the performance of the antenna coil is a critical factor in determining power reception efficiency and data communication reliability. However, as sensors become smaller, reducing coil size drastically reduces communication sensitivity, making it crucial to design a coil that delivers optimal performance within a limited area. This study focuses on the optimal design of a miniature antenna coil for a wearable sensor capable of measuring skin hydration. Considering the characteristics of wearable devices, the design and experimental validation were conducted to maintain a stable resonant frequency and robust power reception and data communication even under mechanical deformation, such as bending of the skin surface. Consequently, a compact, battery-free sensor platform integrated with the optimized antenna coil enables real-time monitoring of patient skin hydration, and its durability has been proven through rigorous environmental testing. Furthermore, polydimethylsiloxane (PDMS) encapsulation ensures mechanical durability and long-term stability. This study highlights the importance of coil optimization in the development of next-generation wearable healthcare devices and provides a basic design framework for miniature sensor systems.

**Supplementary Information:**

The online version contains supplementary material available at 10.1038/s41598-026-38764-5.

## Introduction

Wearable technology is rapidly evolving, enabling real-time health monitoring, autonomous health management support, and early disease prevention^1^. These devices, designed to respond immediately to changes in biosignals, enable personalized health management and present a new medical paradigm^2–10^. However, one of the most fundamental technical challenges for flexible wearable sensors is securing a stable power supply. Currently, most wearable devices still rely on large, rigid batteries or other energy storage devices, hindering sensor miniaturization and limiting user mobility.

To overcome these limitations, the development of ultra-thin sensors utilizing flexible nanomaterials is actively underway^11–16^. Current research focuses on detecting a wide range of biosignals, including electrocardiograms, cardiovascular activity^17,18^, oxygen saturation, body temperature^19,20^, and sweat moisture^21–26^. These sensors have been applied to diverse user groups, including newborns^27^, healthy adults, and wheelchair-bound rehabilitation patients^28^.

Near-field communication (NFC) is another key technology attracting attention. NFC, operating at 13.56 MHz, enables real-time data transmission and energy harvesting simultaneously through inductive coupling, enabling lightweight, compact form factors without the need for bulky external batteries^29^. Recently, it has been successfully demonstrated in biodegradable brain implants, demonstrating reliable and precise wireless performance in both skin-mounted and implantable systems^30^. NFC-based magnetic field control has also been applied to drug delivery systems, enabling iontophoresis- and electrodialysis-based drug release^31^. However, applying NFC to small, flexible sensors remains a challenge. The performance of these systems is determined by the efficiency of the antenna coil responsible for wireless power transfer (WPT) and data communication. Miniaturization is essential for wearable devices, but it directly limits coil performance. Reducing coil size significantly reduces communication sensitivity and power reception efficiency. Therefore, a highly efficient coil design is necessary to achieve optimal performance within a limited space^32,33^.

Furthermore, wearable sensors attached to the body undergo continuous mechanical deformation, such as bending and twisting along the skin contours^34,35^. These variations change the geometry of the coil, causing mistuning (changes in inductance and resonant frequency), which can severely reduce power transfer efficiency and lead to data errors. To ensure long-term reliability in practical applications, the coil design must be robust against mechanical stress. Accordingly, mechanically stretchable circuit designs, such as serpentine interconnects or liquid-metal-based conductors, have been explored as effective strategies to mitigate deformation-induced mistuning in wearable and stretchable electronic systems^36^.

In addition to explicit serpentine geometries, circular antenna coils have also been shown to accommodate mechanical deformation through in-plane bending and elliptical transformation under strain, providing stable NFC operation without complex stretchable interconnect designs^37^.

In skin-attached sensor applications, PDMS encapsulation is commonly employed as a packaging material that provides sufficient flexibility to accommodate physiological skin strain (≤ 15%). Owing to its low elastic modulus and excellent mechanical compliance, PDMS can conform closely to the curved surface of the skin, while its chemical and thermal stability, as well as biocompatibility, have led to its widespread use as an encapsulation material for skin-mounted and wearable electronic devices^38^. Despite these advantages, existing non-invasive sensors may cause discomfort or skin irritation during prolonged wear, and restricted airflow between the skin and the device can result in heat and moisture accumulation, potentially leading to rashes and inflammation. To address these limitations in long-term wearable applications, recent studies have actively explored structurally porous designs that enable moisture drainage and skin respiration^39–41^, along with encapsulation strategies aimed at suppressing corrosion induced by moisture infiltration^42^.

Meanwhile, porous PDMS encapsulation structures enhance breathability and wearing comfort. However, when mechanical deformations such as tensile strain or bending are applied, geometric changes accompanied by variations in the effective dielectric permittivity may occur, which can potentially influence the impedance matching and electrical characteristics of NFC antennas. Therefore, the strain-dependent dielectric behavior should be theoretically considered. Nevertheless, previous studies have reported that porous PDMS behaves as a volume-averaged composite dielectric due to the presence of air voids (Ɛ ≈ 1) distributed within the silicone matrix. As a result, the effective permittivity of porous PDMS remains lower than that of conventional bulk PDMS, and the magnitude of permittivity variation under mechanical deformation is relatively limited^43,44^. Consequently, under low-strain conditions relevant to practical skin-mounted applications, changes in electromagnetic performance are understood to be more dominantly governed by geometric deformation of the antenna coil rather than by strain-induced dielectric modulation.

This study focused on the optimized design of a miniature NFC antenna coil for a wearable moisture monitoring sensor, addressing the complex technical challenges of miniaturization, mechanical deformation, and wearability. The coil structure was systematically designed and experimentally verified to maintain high power transfer efficiency within a limited size while ensuring stable resonant frequency and communication performance even under mechanical stresses such as bending and torsion. Furthermore, by applying a high-power antenna (FEIG Antenna), a wide wireless coverage of tens of centimeters was achieved, suggesting potential for expansion into diverse applications.

The optimized coil was successfully integrated into a battery-free skin hydration sensor platform based on the ISO 15693 protocol. Hydration levels are a key indicator of metabolism and overall body condition and can be measured through sweat analysis. The sensor uses a 14-bit sigma-delta ADC (Analog-to-digital converter) to precisely detect subtle changes. The entire system is encapsulated in flexible, biocompatible porous PDMS (polydimethylsiloxane), providing mechanical durability and excellent breathability, ensuring user comfort even during extended wear.

Previous studies have demonstrated that skin-interfaced sensing systems can maintain stable electrical performance over extended monitoring periods through appropriate selection of electrode materials and encapsulation strategies^42,45^. Based on these findings, the present study employed chemically stable gold (Au) plating to minimize oxidation, corrosion, and signal degradation caused by sweat and skin residues during prolonged wear under direct skin-contact conditions.

In conclusion, this study demonstrates that a geometrically optimized and mechanically robust coil design enables stable operation of a small, battery-free wearable platform in real-world environments. This approach has potential applications in areas such as personalized exercise recovery and chronic disease management, highlighting the potential of next-generation wearable sensors in practical health monitoring applications.

## Results

### Skin-attachable and biocompatible NFC battery-free moisture sensor system

**Fig. 1:**
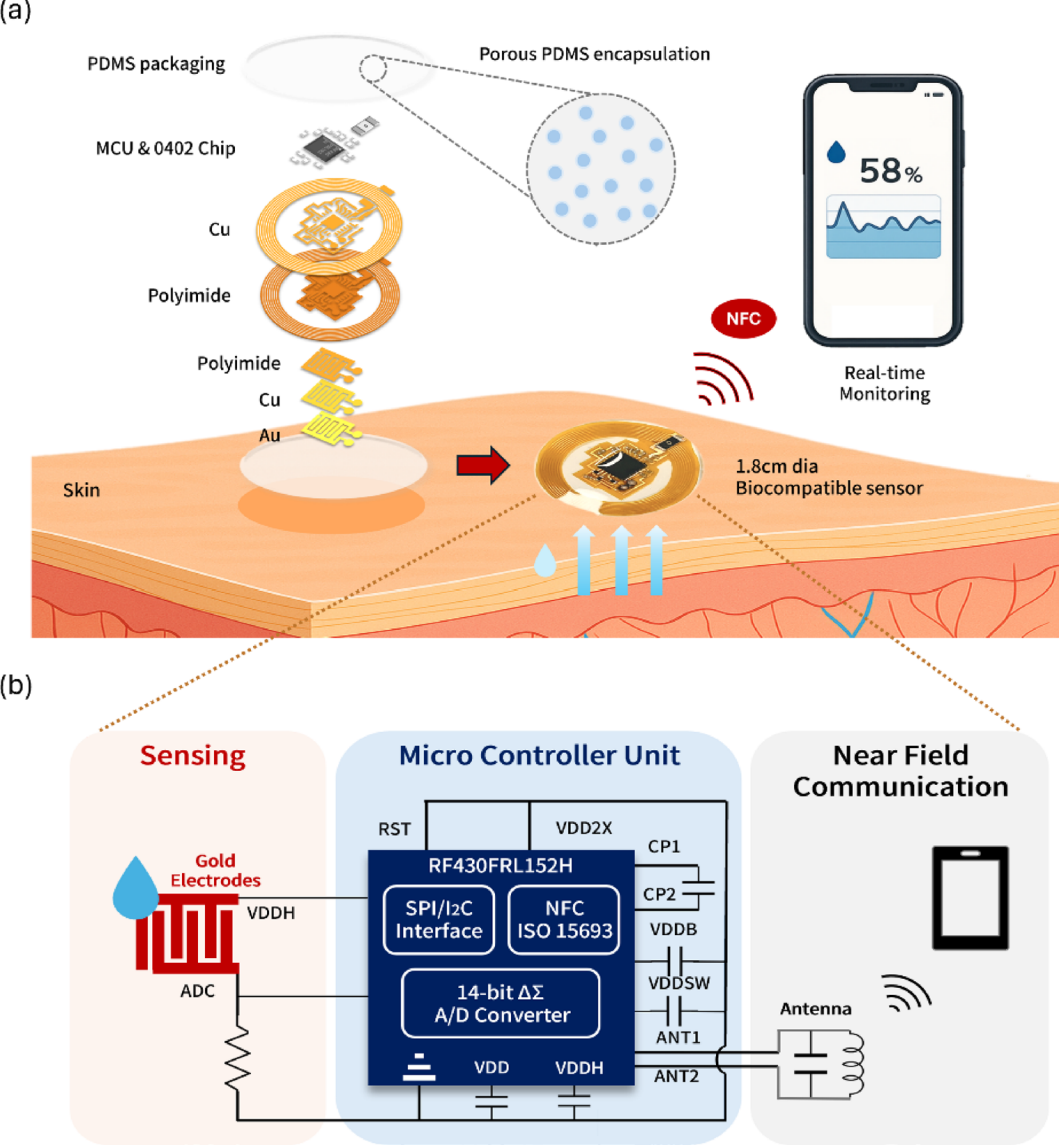
Schematic illustration of an NFC-based battery-free skin hydration monitoring sensor platform. (**a**) System overview of the 1.8 cm biocompatible sensor attached to skin, with real-time hydration data transmission to a smartphone via NFC. (**b**) Detailed circuit diagram of the sensor, including gold electrodes, sensing unit, ADC, NFC interface, and antenna coil.

Figure 1a shows an exploded view and operational environment of a battery-free epidermal moisture monitoring patch. The device is based on an FPCB platform featuring a multilayer structure with copper interconnects connected by microvias. Leveraging the inherent mechanical flexibility and thinness of FPCB, the system demonstrates excellent mechanical resilience, a minimized form factor, and robust electrical stability even under repeated bending and torsional deformation. The bottom layer incorporates gold-plated electrodes, providing a stable skin-electrode interface and ensuring long-term signal acquisition without oxidation. The entire assembly is encapsulated within a microporous PDMS matrix. PDMS is known for its biocompatibility, mechanical flexibility, and chemical inertness, enabling the development of skin-friendly epidermal electronics. The introduction of a microporous structure makes the encapsulation breathable and lightweight, mitigating heat and moisture accumulation during prolonged wear while imparting hydrophobicity to enhance operational durability. Furthermore, the porous structure demonstrates enhanced flexibility. Internally, the integrated circuit platform seamlessly integrates miniaturized surface-mount components, including an NFC-enabled analog front-end chip (RF430FRL152H, Texas Instruments) with integrated ADC/DAC(Digital-to-Analog Converter) functionality and 0402-grade passive components, ensuring robust and reliable electronic performance. This integrated moisture sensor easily attaches to the skin without adhesive and measures moisture levels via unpackaged, comb-shaped gold electrodes. The attached sensor is thin, lightweight, and flexible, adapting well to the dynamic elastic properties of skin. As a result, the sensor is thin and light (approximately 1.8 cm in diameter and approximately 1 mm thick), ensuring close contact and consistent conformal contact with the skin. Furthermore, its high flexibility and adhesion ensure perfect adhesion to contoured or dynamically moving skin surfaces, reducing discomfort even during prolonged use. Furthermore, its compact size and light weight make it ideal for continuous monitoring of wearable sensors, allowing for natural body movements without restrictions. Tagging the attached sensor with an NFC-enabled device allows wireless data transmission for real-time monitoring. Figures 1b is functional block diagrams illustrating the operating principle and electronic architecture of the moisture sensor. An integrated NFC chipset controls both wireless energy harvesting and bidirectional data transmission via the ISO15693 protocol. The analog input from the gold-plated sensing electrode, which quantifies skin moisture through resistive modulation within a voltage divider network, is digitized and processed in real time by an on-chip 14-bit sigma-delta ADC. This platform is applicable to any skin area requiring moisture monitoring, not just the arm, and provides a wireless sensing solution capable of continuous battery-free operation in various biomedical and wearable applications. Moisture data collected wirelessly via an optimized antenna coil is shown in Fig. 4, demonstrating its adaptability to various environments and the stability and reliability of the signal.

### Power transfer efficiency according to coil characteristics for optimized wireless communication

**Fig. 2:**
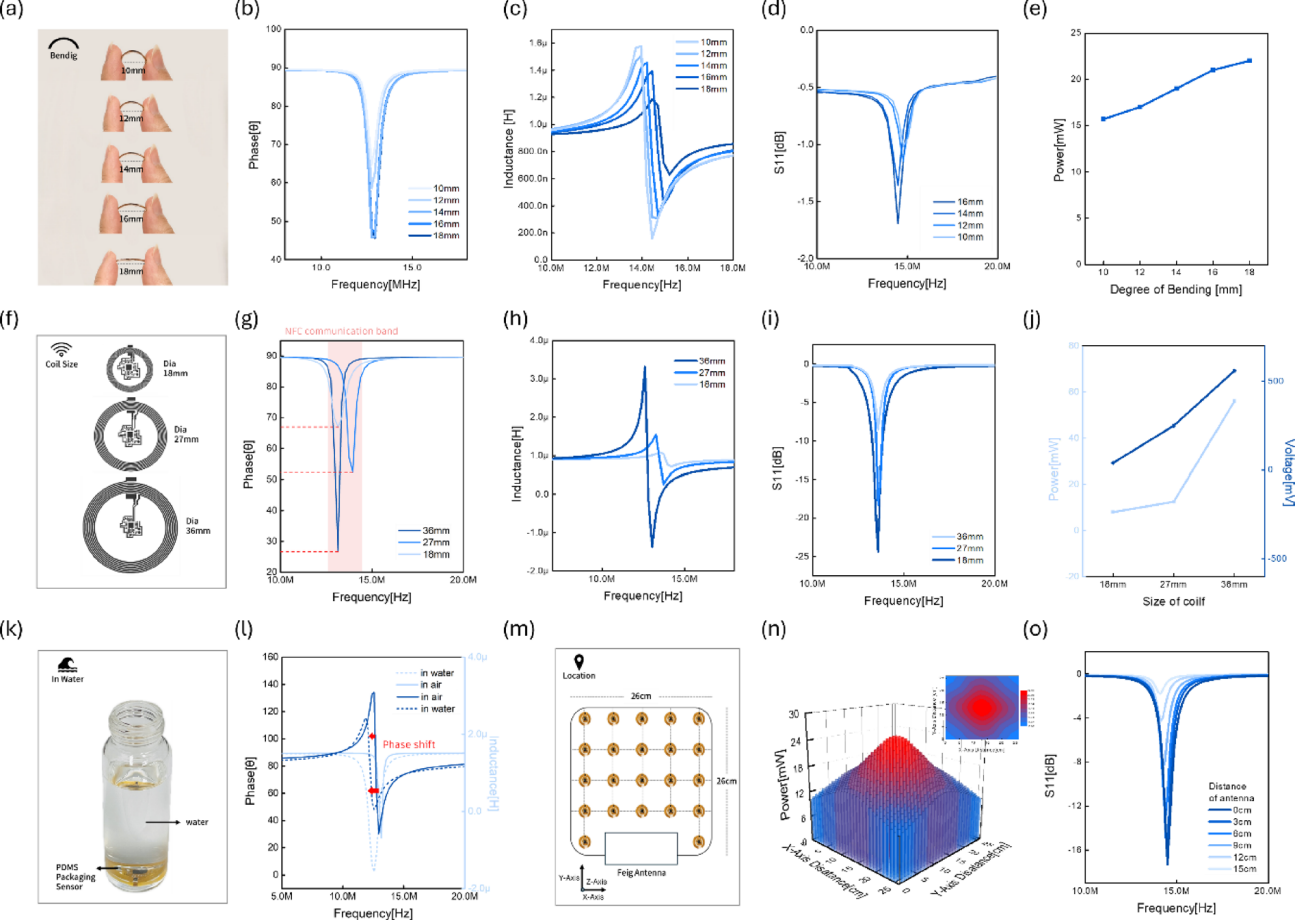
Comprehensive analysis of the mechanical, electrical, waterproof, and wireless power transfer performance of the NFC sensor system. (**a**) Photographs of the sensor being gradually bent by hand (radius: 1.8–1.0 cm), simulating the sensor’s flexibility. (**b**–**d**) Electrical characteristics as a function of frequency under various bending conditions (1.0–1.8 cm): (**b**) phase angle, (**c**) inductance. The peak occurs at the resonant frequency of 13.56 MHz. As the sensor is bent, the electrical characteristics weaken and the resistance increases, but it remains sufficiently high within the communication range. (**d**) Reflection coefficient (S11) graph as a function of bending. (**e**) Wireless power transfer when the sensor is bent. As the sensor is bent (the diameter decreases), the resistance increases and the transmitted power decreases, but it is sufficient for data communication. (**f**) Schematic diagram of the antenna coil geometry (baseline: 18 mm, zoom: 27 mm and 36 mm): This shows the wireless power transfer as a function of antenna size. (**g**–**h**) Frequency response of the sensor with respect to antenna size: (**g**) phase, (**h**) inductance: As the coil diameter increases, the magnetic flux increases and the resistance increases. (**i**) Reflection coefficient (S11) plotted using a network analyzer with respect to coil size: The value increases with increasing diameter, confirming the decrease in input signal absorption. (**j**) Power and voltage values ​​wirelessly received by the antenna with respect to coil size. (**k**) Real-time monitoring of the PDMS package sensor in water. (**l**) Frequency vs. phase and impedance response plots in air and water. (**m**) Schematic diagram of the sensor locations in the Feig antenna array (26 × 26 cm) for evaluating spatial power distribution. (**n**) A 3D surface plot showing the wireless energy reception power distribution in the X-Y plane and a 2D contour color fill plot representing the same data with a contour-based color gradient. (**o**) Variation of reflection coefficient (S11) with respect to the sensing distance between the sensor coil and the Feig antenna: showing the effect of spatial separation on impedance matching and coupling efficiency. * All experimental data shown in this figure were obtained using the fully integrated sensor with the antenna coil.

Figure 2 shows the results of various experiments aimed at optimizing wireless power. In particular, the NFC communication-based sensor used in this paper wirelessly receives power from the coil, making coil characteristics crucial. Therefore, coil characteristics (such as the number of turns, coil spacing, and coil wire thickness) affect power transfer efficiency. This study aims to design a unique antenna that contributes to stable communication while minimizing coil size through an optimized design.

First, to test mechanical durability and flexibility, the sensor was bent from a diameter of 1.8 cm to a radius of 1 cm, as shown in Fig. 2a, and the electrical characteristics and power efficiency at each bending stage were investigated. Sequential images confirmed that the sensor maintained its structural integrity without any noticeable damage even with progressive bending, demonstrating its suitability for wearable devices attached to the skin. In-depth analysis of frequency-dependent electrical characteristics was performed for each bending angle. The phase response (Fig. 2b) showed minimal change at 13.56 MHz for bending radii of 10, 12, 14, 16, and 18 mm, with a peak observed at a point where the resonant frequency remained unchanged. The electrical characteristics of the antenna coil region decreased with bending, resulting in a decrease in the phase value. However, the decrease was minimal and did not affect communication. Similarly, the inductance curve (Fig. 2c) showed a slight increase with bending, but the resonant frequency remained stable, confirming stable electrical performance. Figure 2d shows the reflection coefficient S11 graph, which confirms the decrease in reflection coefficient with bending. The wireless power reception graph with bending (Fig. 2e) shows that the received power decreased slightly with decreasing bending radius due to geometric detuning of the antenna coil within the integrated sensor, but maintained a sufficient power level for stable sensor operation. Consequently, as the sensor was bent (the sensor diameter decreased), the effective magnetic coupling and resistance of the antenna coil within the integrated sensor changed, resulting in a decrease in the phase response and a decrease in the received power. However, this range still enables sufficient NFC communication and data transfer.

To determine the optimal coil geometry for NFC-based wireless power transfer, coils with diameters of 18 mm, 27 mm, and 36 mm were fabricated (Fig. 2f). The frequency-phase and frequency-inductance graphs (Fig. 2g and h) show that the 18 mm coil exhibited the lowest inductance and phase shift despite its smallest size, demonstrating minimal energy loss and high magnetic coupling efficiency. Inductance is proportional to the coil area and the square of the number of turns, but as the coil size increases, efficiency decreases due to increased parasitic resistance and stiffness.

The reflection coefficient S11 (Fig. 2i) measured with a network analyzer showed the deepest resonance dip of approximately − 20 dB, demonstrating the best impedance matching and power coupling performance. Although the output power and voltage tend to increase with increasing coil diameter (Fig. 2j), the 18 mm coil, despite being an ultra-small sensor, simultaneously ensured sufficient power transmission capability and excellent flexibility. Therefore, the 18 mm coil was selected as the optimal design to satisfy both performance and miniaturization of NFC-based sensors.

NFC-based wireless power transfer utilizes magnetic coupling at 13.56 MHz high frequency (HF) to form a long wavelength, enabling stable communication even underwater. Therefore, electromagnetic characteristics and communication performance do not significantly degrade even when attached to the skin, which is advantageous for expanding into implantable sensors.

To verify this, the sensor was packaged in PDMS, as shown in Fig. 2k, and its electrical characteristics were measured after being fully submerged in water. The frequency–phase and inductance–frequency graphs (Fig. 2l) show that the resonant frequency only slightly shifts within the communicable range even in an underwater environment, confirming that stable wireless power transmission is possible even underwater. When the sensor is immersed in water, the surrounding medium changes from air (relative permittivity ≈ 1) to water (relative permittivity ≈ 80). This increases the permittivity of the surrounding medium, which in turn increases the capacitance and decreases the resonant frequency, causing the graph to shift to the left. Nevertheless, the resonant frequency remains within a stable communicable band, demonstrating that reliable wireless communication is possible even underwater.

Furthermore, the spatial distribution of wireless power was measured using a 26 × 26 cm FEIG antenna array (Fig. 2m). The 3D power mapping (Fig. 2n) confirmed that maximum coupling occurred at the center of the antenna and gradually decreased toward the edges. This demonstrated uniform and stable wireless power reception across the entire operating range.

To evaluate the power transfer efficiency as a function of distance between the FEIG antenna and the sensor, the reflection coefficient (S11) was analyzed while varying the coil distance from 0 cm to 15 cm (Fig. 2o). While the resonance dip depth tended to decrease with increasing distance, sufficient coupling efficiency and stable power transfer were maintained within approximately 15 cm.

### Experiments on porous PDMS packaging with improved durability and flexibility

**Fig. 3:**
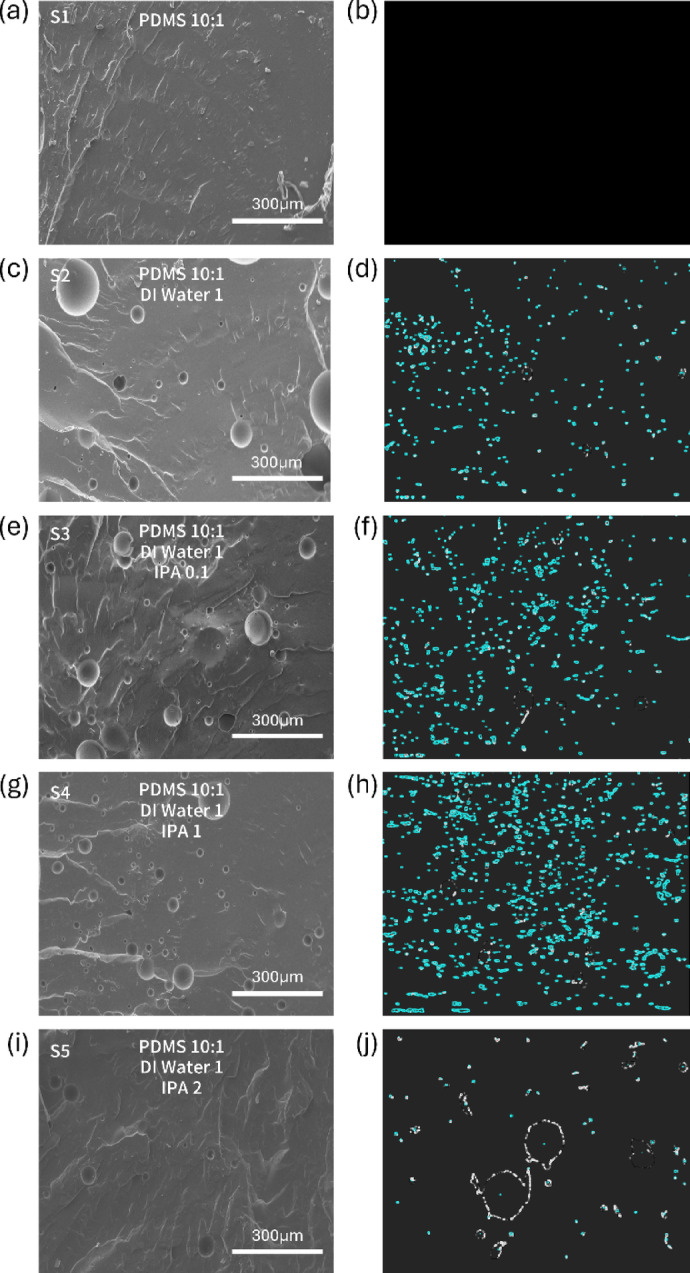
SEM images of five samples showing the porosity distribution according to the mixing ratio of the materials. (**a**) Mixture of pdms base 10 g+pdms agent 1 g. (**b**) Pore analysis image of (**a**). (**c**) Mixture of pdms base 10 g+pdms agent 1 g+DIwater 1 g. (**d**) Pore analysis image of (**c**). (**e**) Mixture of pdms base 10 g+pdms agent 1 g+ DIwater 1 g + IPA 0.1 g. (**f**) Pore analysis image of (**e**). (**g**) Mixture of pdms base 10 g+pdms agent 1 g+ DIwater 1 g + IPA 1 g. (**h**) Pore analysis image of (**g**). (**i**) Mixture of pdms base 10 g+pdms agent 1 g + DIwater 1 g + IPA 2 g. (**j**) Pore analysis image of (**i**).

**Fig. 4:**
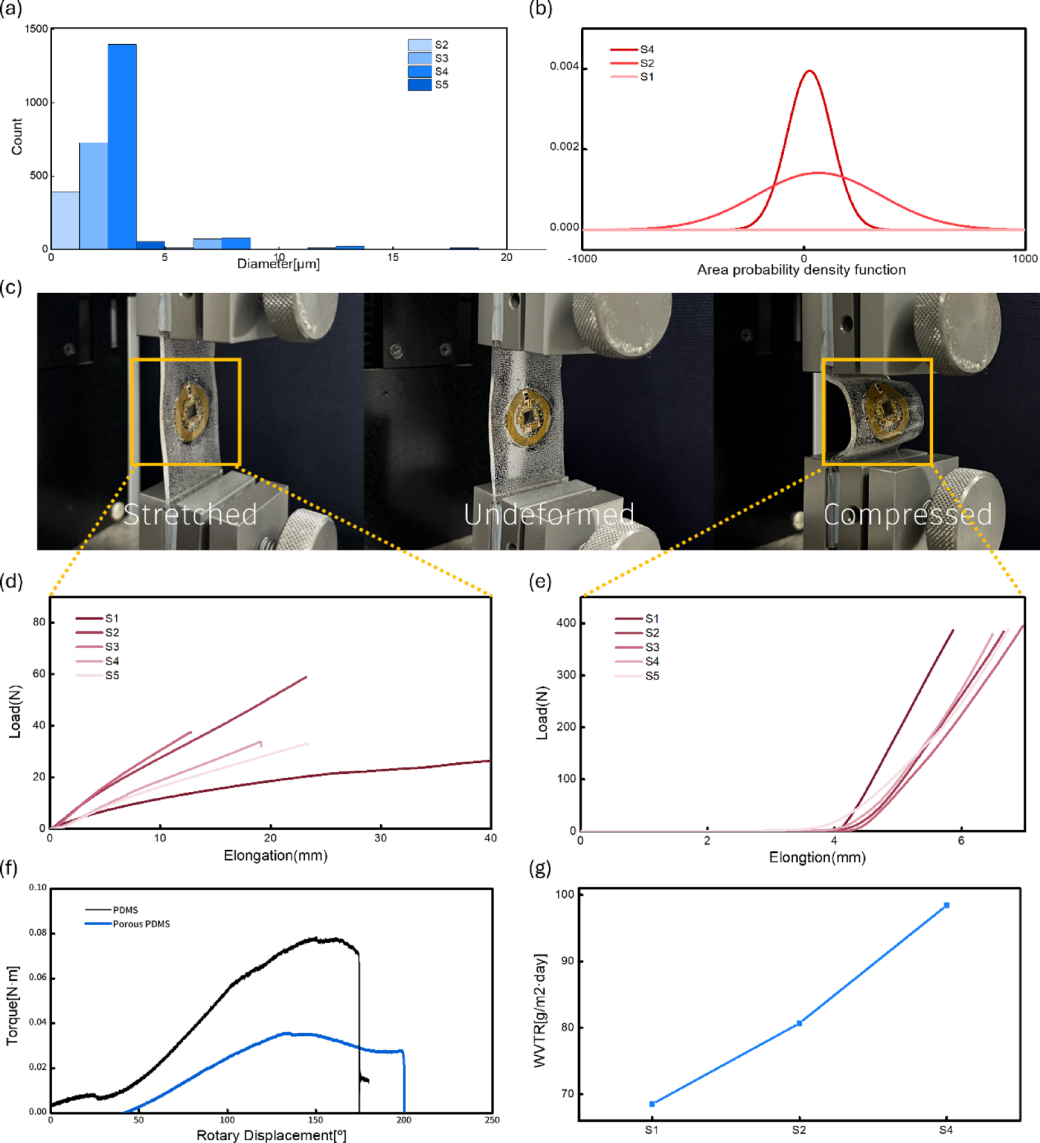
Characterization of porous PDMS encapsulation. (**a**) The graph shows the area uniformity of pores that varies depending on the mixing ratio of the materials. (**b**) The area distribution characteristics of pores according to the mixing ratio were expressed in the form of a probability density function, and the results were compared to see which composition formed the most uniform pore structure. (**c**) Actual porous encapsulation sensor testing, including tensile and compression tests. (**d**) Tensile test results with and without pores. (**e**) Compression test results with and without pores. (**f**) Torsional test results with and without pores. (**g**) Water vapor transmission rate test results and the number of pores formed according to the presence and absence of pores. *All experiments were performed 30 times to ensure statistical reliability.

Wearable sensors must ensure durability, ease of use, biocompatibility, and wearer comfort during long-term use. However, one of the major limitations of patch-type devices is poor breathability, which can cause skin irritation and discomfort, ultimately leading to performance degradation. To address this issue, this study developed a flexible and breathable polydimethylsiloxane (PDMS) packaging film with enhanced porosity. This film enhances moisture permeability while maintaining mechanical stability.

PDMS is a transparent, flexible, and biocompatible material with both viscous and elastic properties, making it widely used in wearable electronic devices. However, its poor breathability limits long-term wearability. In this study, micropores were formed by mixing diluted distilled water (DIW) and isopropyl alcohol (IPA) into PDMS. Water droplets formed during the mixing process evaporate during the curing process, forming uniform pores. Cross-sectional SEM images of the fabricated sample confirmed the formation of a uniform and distinct pore structure compared to conventional non-porous PDMS (Fig. 3). Furthermore, quantitative analysis of pore size and distribution according to the mixing ratio (Fig. 4a) revealed that varying the DIW and IPA ratio systematically controlled the pore uniformity and area fraction.

Figure 4b, which quantitatively analyzes the uniformity and distribution characteristics of the pore size formed within each sample, shows the area distribution of pores generated according to the mixing ratio in the form of a probability density function. The x-axis represents the pore area, and the y-axis represents the probability density of the corresponding area. The width and height of the curve reflect the uniformity of the pore distribution. Therefore, this graph, with its narrow and sharp distribution, is the most uniform and confirms the formation of a large number of micropore structures.

Next, tensile, compression, and torsional tests were performed to evaluate the effect of pore formation on mechanical properties (Fig. 4c–f). As a result, the porous PDMS film exhibited superior flexibility, with lower stiffness and higher elongation compared to the non-porous PDMS film. In particular, the torsion test results (Fig. 4f) demonstrate that the porous film exhibited stable displacement-torque behavior even under dynamic deformation, demonstrating excellent mechanical resilience under bending, stretching, and compression conditions.

Finally, the water vapor transmission rate (WVTR) and pore density were analyzed to evaluate the effect of the porous structure on improving moisture permeability (Fig. 4g). The experimental results showed that the moisture permeability of the porous PDMS film was approximately twice that of the conventional PDMS film.

In conclusion, the porous PDMS packaging developed in this study effectively improves the low moisture permeability of conventional PDMS while ensuring high flexibility and mechanical stability. Therefore, it is expected to be an optimal packaging material for wearable sensor applications requiring long-term wear.

### Real-time sensor response under various condition evaluation

**Fig. 5:**
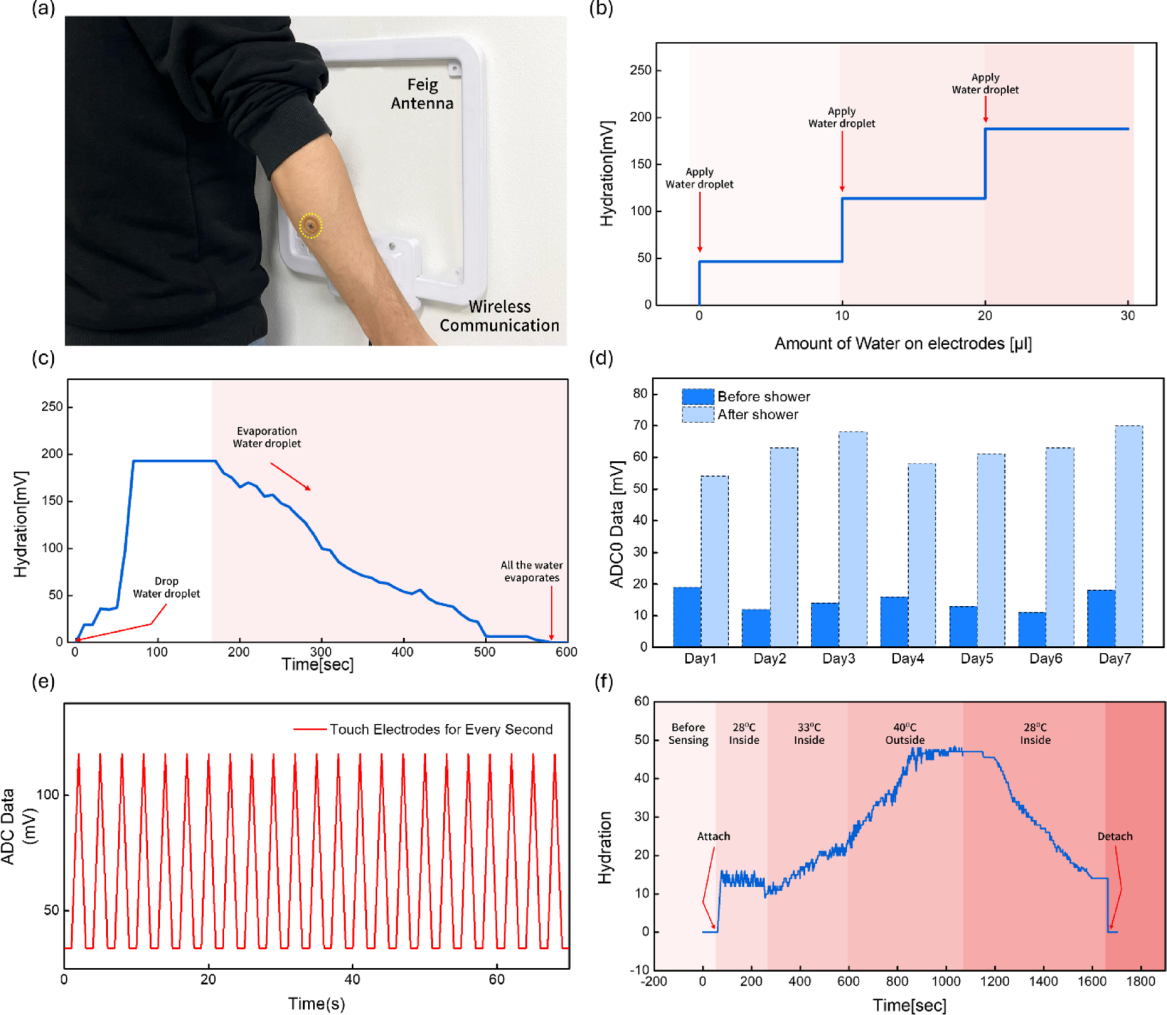
Wireless epidermal moisture sensor performance evaluated through real-time monitoring under various moisture conditions. (**a**) Photograph of the experimental setup demonstrating wireless communication between the skin-mounted sensor and the FEIG HF antenna. (**b**) Graph showing voltage changes after three 10 µL droplets were placed on the electrode. (**c**) Data showing the drying time of water droplets on the electrode over a 10-minute period. (**d**) Graph showing skin moisture levels measured before and after showering while wearing the sensor for 7 days. (**e**) Real-time ADC signals obtained by periodically touching the electrode every second. (**f**) Data showing moisture levels measured on the arm at various temperatures before and after wearing the sensor.

In this study, skin hydration was measured in real time using a wirelessly enabled FEIG antenna (Fig. 5a). The sensor demonstrated stable and reliable data acquisition across diverse environments and measurement modalities. Furthermore, the sensor’s highly durable design ensured long-term use, resulting in highly reproducible data even after several days. These results demonstrate the potential of the sensor as a next-generation wearable platform for reliable battery-free biosignal monitoring, supported by an optimized antenna coil design and stable wireless communication and power reception. Furthermore, by combining a wireless communication-based energy harvesting structure with a flexible design, we laid the foundation for a user-friendly digital healthcare solution that is skin-friendly and requires no maintenance or replacement.

To quantitatively analyze the sensor’s moisture sensitivity, 10 µL droplets were sequentially dropped onto the sensing electrode surface (Fig. 5b). With each droplet added, the detection data gradually increased, demonstrating the sensor’s sensitivity to moisture content changes and high resolution. These results demonstrate that the precisely designed comb-shaped electrode structure effectively amplifies moisture-induced permittivity changes, enabling the sensor to precisely detect even subtle moisture changes. To evaluate the dynamic hydration monitoring performance, we dropped a water droplet onto the electrode and observed changes in the detection signal during the evaporation process (Fig. 5c). As the droplet evaporated, the sensor output gradually decreased over approximately 10 min, mimicking a process similar to actual transepidermal water loss (TEWL). The sensor’s ability to track hydration loss in real time suggests its potential for dynamic physiological signal monitoring. To verify long-term reliability and repeatability in a real-world environment, the wearer attached the sensor to their wrist for 7 days and recorded daily hydration measurements using an NFC reader before and after showering (Fig. 5d). Immediately after showering, the ADC output value increased significantly as skin surface moisture increased, and this phenomenon persisted throughout the entire measurement period. Notably, the sensor maintained stable detection performance without mechanical damage even when exposed to humid environments or external water. This is attributed to the waterproof and moisture-permeable properties of the porous PDMS packaging structure, as shown in Fig. 2. These results demonstrate the long-term stability of this sensor in real-world environments. Figure 5e shows the sensor response when the electrodes are touched at 1-second intervals. The ADC output exhibits an immediate rise and decay pattern with each stimulus. This demonstrates that the sensor exhibits high temporal resolution in response to external stimuli and maintains a consistent signal pattern even with repeated contact.

Furthermore, to verify the sensor’s sensitivity to environmental interference, the ADC response was measured before and after skin attachment and under various temperature conditions (indoor 28 °C, indoor 33 °C, outdoor 40 °C) (Fig. 5f). The pre-skin attachment measurements remained virtually unchanged in the outdoor environment, indicating that error signals due to external temperature and humidity were minimized. In contrast, after the sensor was attached to the skin, the moisture signal increased rapidly as the indoor temperature increased, reflecting sweating on the arm. This suggests that the sensor can sensitively track physiological moisture changes in the human body. Furthermore, as shown in the moisture evaporation data in Fig. 5c, when the patch was attached to actual skin, the moisture content decreased as the environment changed from a hot outdoor environment to a cool indoor environment. These results demonstrate that the proposed sensor is capable of real-time, wireless, high-precision moisture monitoring and operates reliably in changing external environments. In particular, the differential response before and after skin attachment and the dynamic moisture detection performance depending on temperature demonstrate that the sensor can sensitively track skin physiological changes. Combining a power-free NFC-based actuation structure and porous PDMS packaging technology, this sensor can be utilized as a skin-attached digital healthcare device that can be worn for extended periods of time in daily life and has the potential for a wide range of applications, including personalized skin care, post-exercise recovery monitoring, and skin moisture management for patients with chronic diseases.

## Discussion

In this study, we developed a battery-free, skin-attached moisture monitoring sensor that integrates an NFC antenna coil optimized for miniaturization and wearability, enabling both stable wireless communication and efficient power transfer. The precisely engineered, flexible FPCB-based circuit design ensured stable electrical characteristics and reliable signal detection even when placed in close contact with the skin.

Importantly, the optimized antenna coil maintained a stable resonant frequency at 13.56 MHz and exhibited consistent electrical performance even under repeated mechanical deformations such as bending, twisting, and sweat exposure. These results demonstrate the critical role of antenna coil design in addressing the dual challenges of miniaturization and mechanical reliability in wearable NFC platforms.

Furthermore, we developed a patch-type sensor that facilitates long-term monitoring by ensuring mechanical durability and breathability through porous PDMS encapsulation using biocompatible materials. Compared to existing wired devices or bulky impedance-based systems, the proposed sensor is significantly lighter, more flexible, and more user-friendly, resulting in superior wearability. In real-world experiments, this device accurately and reliably detected skin moisture changes and consistently tracked moisture changes caused by natural perspiration in high-temperature environments.

This demonstrates its potential for applications that provide user-centric, time- and space-independent next-generation monitoring digital healthcare. Furthermore, future research can leverage the electrical characteristics of wireless communication to develop an implantable sensor platform. In addition, future studies may explore mechanically stretchable circuit architectures, such as serpentine interconnects or liquid metal conductors, to further extend the applicability of the proposed platform to scenarios involving larger or more complex deformations, including joint-mounted or highly dynamic wearable applications.

## Materials and methods

Fabrication of FPCB Battery-free Sensor for Hydration Sensor (Supporting Information Fig. S1): The single-sided FPCB (18 μm, 1/2 OZ) was cut to match the circuit-printed mask size and subjected to dual-side cleaning using isopropanol (IPA) and N₂ gas. The circuit was then printed on the flexible copper (Cu, 18 μm)/polyimide (PI, 25 μm) film (FPCB, SME Trading Co., Ltd., Korea) by photolithography. The photoresist (AZ4620, AZ Electronic Materials, Darmstadt, Germany) was coated to an 8 μm thickness on the film using a spin coater (LT-MS 150, LTS, Seongnam, Korea) at 3,000 rpm for 40 s. After coating, the film was softly baked on a hot plate (HSD150, MTOPS, Yangju, Korea) at 110 °C for 30 s. The cleaned film was placed on the central chuck of the spin coater and securely fastened with a vacuum. Photo Resist (PR) (1.0–1.5 ml) was carefully dispensed onto the film’s center using a syringe to prevent air bubbles. Spin-casting was conducted at 4,000 rpm for 40 s after activating the vacuum pump. Soft baking was performed on a hot plate at 110 °C for 1 min and 30 s. After cooling to room temperature, the film was placed on the mask aligner, aligning the photomask with the substrate and ensuring that the PR-coated side faced upwards using the lever. The film was then adhered to the photomask and assisted in contact by turning on the vacuum. For custom geometry patterning of the designed circuit, photolithography (COOLUV-100, JSE, Anyang, Korea) was performed at a UV irradiance of 10 mW cm⁻² for 2 min. The unpatterned area was removed using a developer (AZ 300 MIF developer, AZ Electronic Materials Ltd., KOR). After developing, hard baking was carried out at 110 °C for 1 min, and the copper sheet was wet-etched to fit the circuit pattern using a CE-100 copper etchant (Transense, Bicester, UK) at 70 °C. Once activated (40–60 °C), the film was inserted and left for 1–1.5 min for etching. Following etching, cleaning was conducted using DI water, acetone (ACE), IPA, and N₂ gas in sequence to remove the PR layer on the circuit pattern.

Gold plating of the electrodes was performed using an electroless gold plating process. Briefly, the fabricated copper electrodes on the polyimide substrate were immersed in a commercial electroless gold plating solution (Sigma-Aldrich, Product No. 901670) under controlled conditions, enabling uniform deposition of a gold layer on the electrode surface without external electrical bias. After plating, the electrodes were thoroughly rinsed with deionized water and dried prior to further device assembly.

Chip components and Soldering (Supporting Information Fig. S2): The RF430ERL152H (Texas Instruments) chip is used in the center of the sensor for ADC and NFC communication. 0402 was used for the resistor capacitor and 1206 size was used for the jumper (0 ohm) to connect the antenna and electrode. In/Sn soldering paste (52In 48Sn, Indium, Clinton, New York, USA) was heated at 190 °C to enable soldering of passive devices, and circuit verification was performed under a microscope to ensure accuracy.

Design Parameters of the NFC Antenna Coils: To ensure experimental reproducibility and enable direct comparison among different coil sizes, the geometric parameters of all NFC antenna coils were explicitly defined. All coils consisted of 8 turns and were fabricated using copper conductors with a uniform thickness of 18 μm. The trace width and inter-turn spacing were symmetrically designed for each coil diameter as follows: 0.15 mm/0.15 mm for the 18 mm coil, 0.23 mm/0.23 mm for the 27 mm coil, and 0.34 mm/0.34 mm for the 36 mm coil. These parameters were selected to maintain consistent fabrication constraints while systematically varying the coil diameter to evaluate its effect on wireless power transfer efficiency.

Fabrication of pore structure in PDMS (Supporting Information Fig. S3): For the PDMS layer, the PDMS (DC-184, DOW CORNING) base and curing agent were mixed in a 10:1 ratio, with 10 g and 1 g respectively, in a beaker using a spatula. If DI water and IPA are mixed, the IPA will produce water droplets. When cured on a hot plate, the water evaporates in the droplets and pores form in their place. DI water was added drop by drop using a pipette to the stirring PDMS mixture, followed by the addition of IPA in the same manner. The beaker was then placed inside a vacuum jar connected to a vacuum pump and left under vacuum for approximately 2 min to remove any trapped air bubbles. After removing bubbles, the PDMS mixture was thinly spread onto a petri dish and cured on a hot plate at 80 °C for 1 h.

Data Collection and Analysis (Supporting Information Fig. S4): The SEM image was analyzed for the number and area of pores using the pore analysis tool in the Image J program: The electromagnetic characteristics were confirmed for NFC communication using an impedance analyzer. Using the GUI software program of the NFC chip provided by TI, the chip can be sensed and the values received from the electrodes in real time can be checked in hexadecimal. Wireless hydration data were collected via the TRF7970A NFC transceiver from Texas Instruments, enabling real-time communication between the sensor and reader. In addition, the S₁₁ reflection coefficient data were measured using the E5063A ENA Vector Network Analyzer to evaluate the impedance matching and RF characteristics of the antenna under various conditions. Real-time hydration measurements were acquired using the FEIG Electronic ID ANT310/310 HF loop antenna (13.56 MHz, up to 8 W output, IP65 rated), which supports reliable NFC communication with read ranges up to 70 cm. This setup enabled stable data transmission between the wearable sensor and the reader in practical measurement environments.

Measurement of Effective Permittivity of Porous PDMS under Tensile Strain (Supporting Information Table S1): The effective relative permittivity of the porous PDMS encapsulation layer under tensile deformation was evaluated using a parallel-plate capacitor model. Porous PDMS samples with a dimension of 20 × 20 × 1.5 mm^3^ (width × length × thickness) were prepared, and silver tape electrodes were attached to the top and bottom surfaces to form a capacitor structure. Uniaxial tensile strain was applied incrementally from 0% to 25% in steps of 5%, while the sample dimensions (effective electrode area *A* and thickness *d*) were recorded at each strain level. The capacitance was measured using a digital LCR meter under ambient conditions. The effective relative permittivity (ε_r_,eff) was calculated from the measured capacitance using the parallel-plate capacitor equation,

$$C = \varepsilon _{0} \varepsilon _{r} \frac{A}{d}$$

where ε_0_ is the vacuum permittivity. The calculated ε_r_, eff and corresponding capacitance values as a function of tensile strain are summarized in Table S1, providing a quantitative assessment of strain-induced dielectric variation in the porous PDMS layer.

Baseline Calibration and Measurement Procedure: Prior to each measurement session, the sensor was detached from the skin and gently cleaned to remove residual sweat or skin contaminants. The device was then activated by an NFC reader in a non-contact (off-skin) condition to acquire a reference signal, which was defined as the intrinsic electronic offset of the sensor excluding the electrode–skin interface effect. This reference value was subsequently subtracted from the signals obtained during on-skin measurements for data analysis, enabling baseline-corrected and drift-minimized long-term monitoring.

Ethical approval and human subject involvement: The demonstration test shown in Fig. 5a involved human participants. All experimental procedures were carried out in accordance with relevant guidelines and regulations. The experimental protocols involving human subjects were reviewed and approved by the Institutional Review Board (IRB) of Kwangwoon University (Approval No. 7001546-202400105-HR(CSB)-012-02). Written informed consent was obtained from all participants prior to their participation in the study.

## Supplementary Information

Below is the link to the electronic supplementary material.

Supplementary Material 1

## Data Availability

Data is provided within the manuscript or supplementary information files.
