# Correction: Miniaturized flexible skin moisture sensor with optimized coil for enhanced wireless power efficiency

**DOI:** 10.1038/s41598-026-45492-3

**Published:** 2026-03-24

**Authors:** Hyejun Kim, Seongu Kim, Changyu Yeo, Minkyung Kim, Weonho Shin, Jeonghyun Kim

**Affiliations:** 1 Department of Electronic Convergence Engineering, Kwangwoon University, Seoul, 01897 South Korea; 2 Department of Electronic Materials Engineering, Kwangwoon University, Seoul, 01897 South Korea

Correction to: *Scientific Reports* 10.1038/s41598-026-38764-5, published online 10 February 2026

Hyejun Kim was omitted from the author list in the original version of this Article.

The original Article has been corrected.

