# Supplementary material for: Miniaturized flexible skin moisture sensor with optimized coil for enhanced wireless power efficiency
Source: Sci Rep. 2026 Feb 10;16:8114. doi: 10.1038/s41598-026-38764-5 (PMC12960965; doi:10.1038/s41598-026-38764-5)
Supplement: Supplementary file 1 — Supplementary Material 1 [file 41598_2026_38764_MOESM1_ESM.docx]

**Supporting Information**

Miniaturized Flexible Skin Moisture Sensor with Optimized Coil for Enhanced Wireless Power Efficiency

Hyejun Kim, Seongu Kim, Changyu Yeo, Minkyung Kim, Weonho Shin*, and Jeonghyun Kim*

H. Kim, S. Kim, and J. Kim*

Department of Electronic Convergence Engineering

Kwangwoon University

Seoul 01897, South Korea

E-mail: [jkim@kw.ac.kr](mailto:jkim@kw.ac.kr)

C. Yeo, M. Kim, W. Shin*

Department of Electronic Materials Engineering

Kwangwoon University

Seoul 01897, South Korea

E-mail: [weonho@kw.ac.kr](mailto:weonho@kw.ac.kr)

**Figure S1. Fabrication process and electroless gold plating procedure of the flexible hydration sensor.**

**
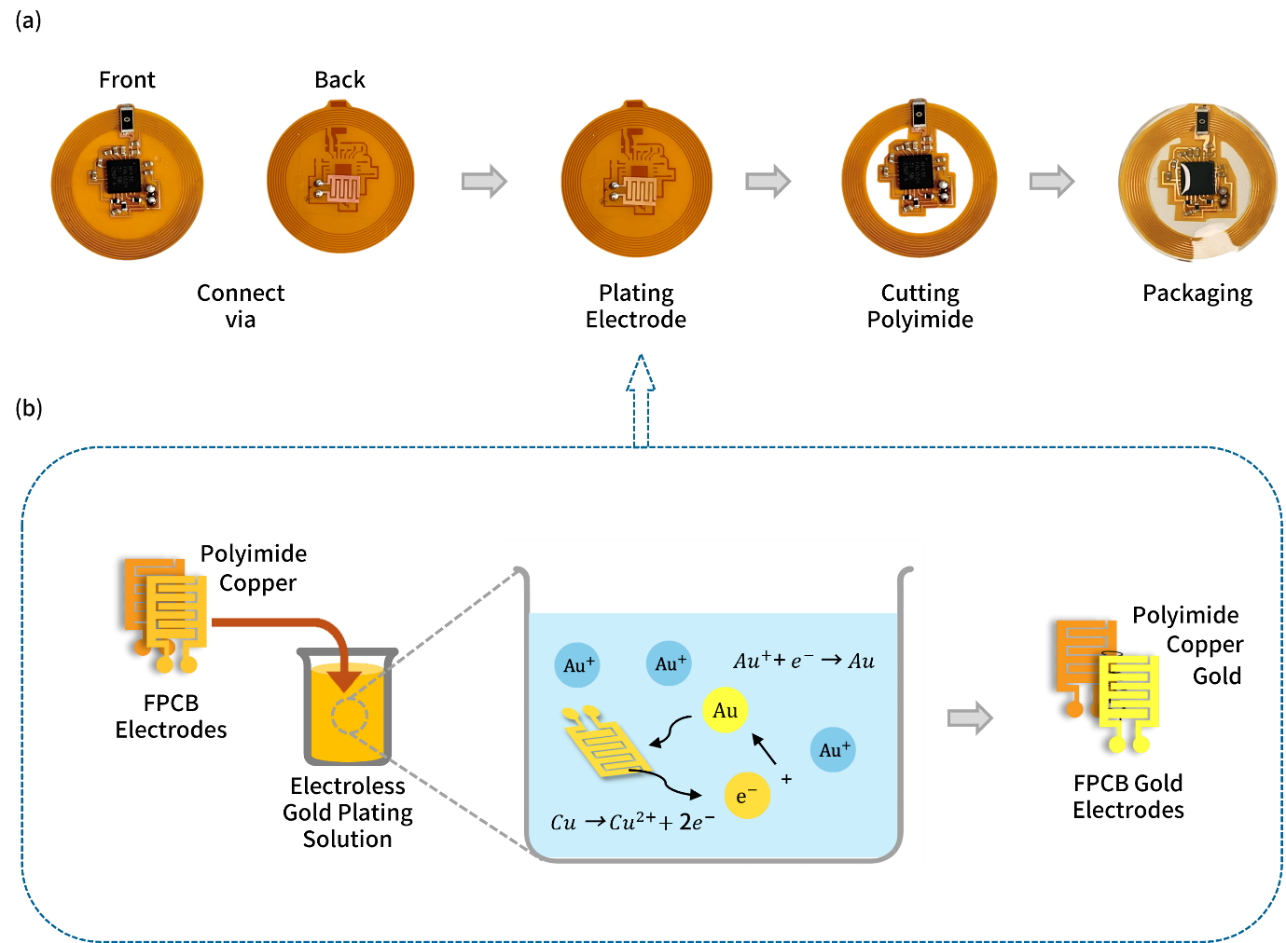
**

(c)

(a) Illustrate step-by-step device fabrication, including via connection between the front and back layers, electrode gold plating, polyimide substrate cutting, and final packaging. (b) Schematic depicts the electroless gold plating process, where the copper electrodes on the polyimide flexible printed circuit board (FPCB) are immersed in a gold plating solution. Gold ions (Au⁺) are reduced by electrons originating from the copper surface, resulting in the deposition of a uniform gold layer onto the electrodes to enhance conductivity and biocompatibility. (c) Electrical resistance of gold-coated interdigitated electrodes measured during continuous water immersion for 14 days, showing no detectable change and confirming the electrochemical stability of the electrode interface.

**Figure S2 Circuit schematic of the FPCB.**


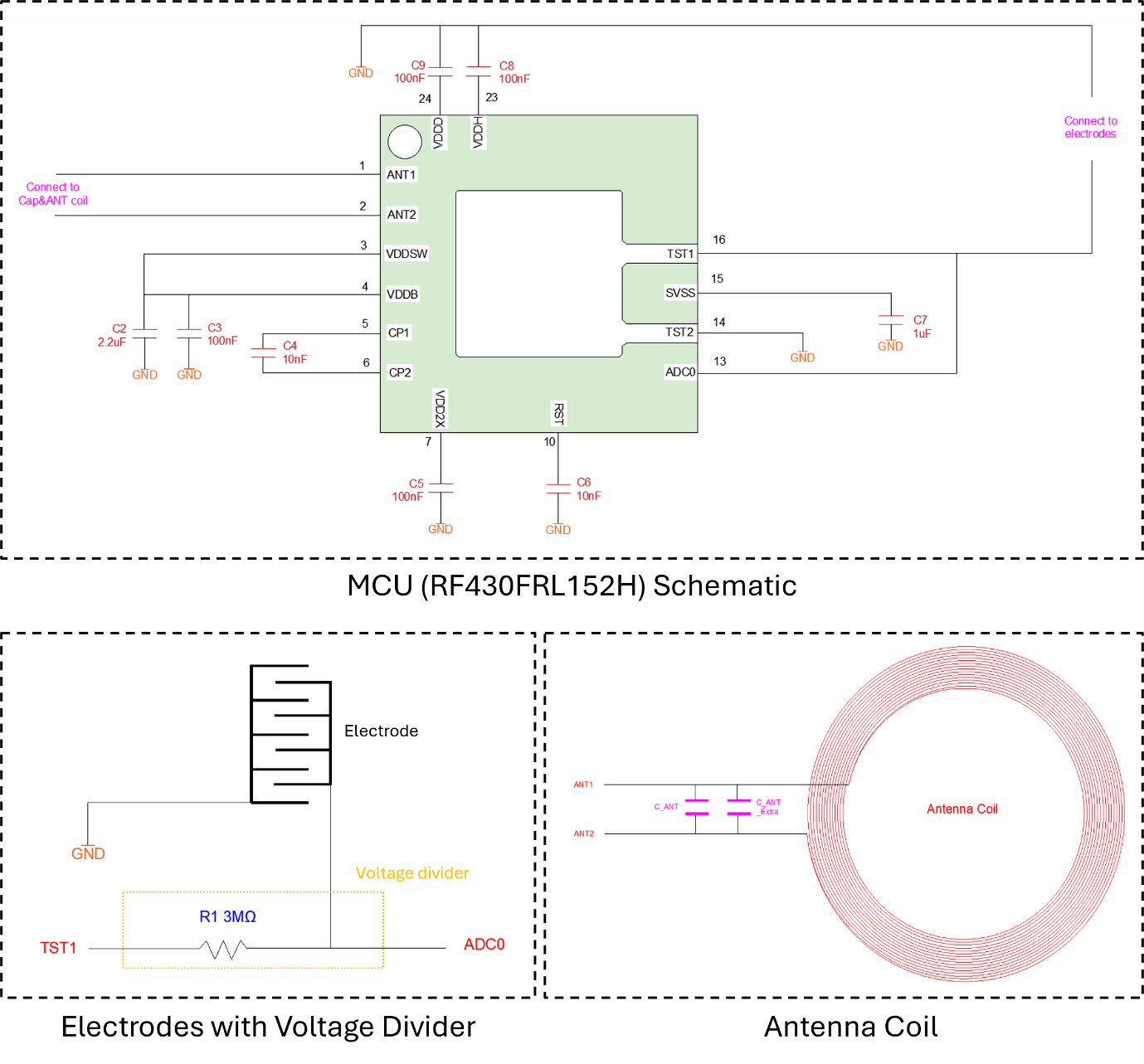


**Figure S3. Fabrication process of porous PDMS films for cross-sectional characterization.**


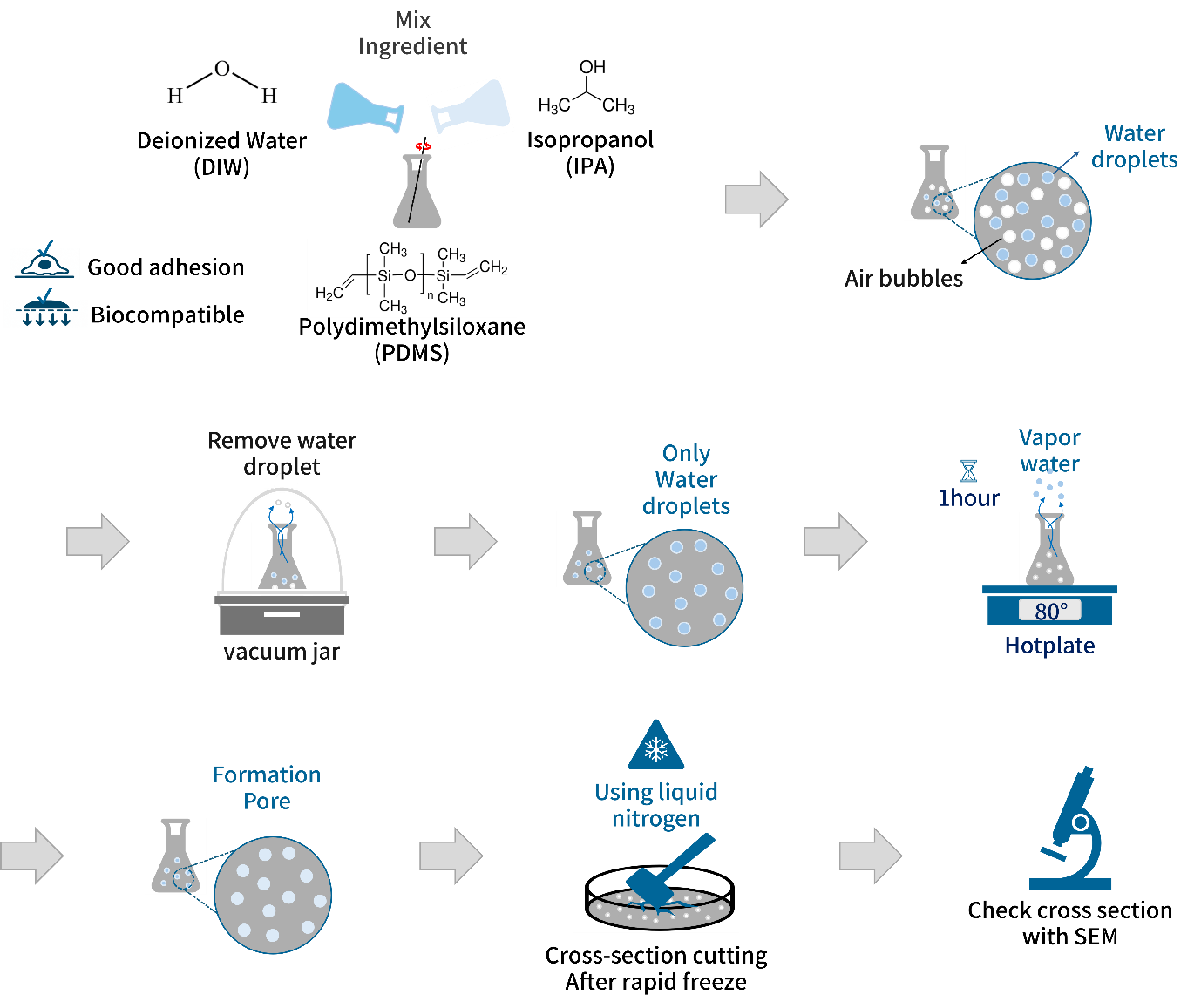


**Figure S4. Electromagnetic characterization of the NFC antenna coil under tensile deformation.**


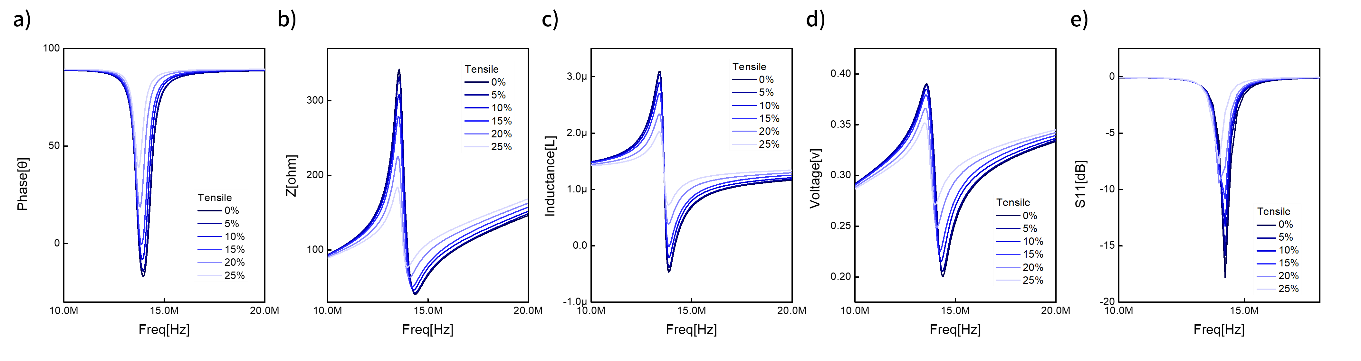

(a) Phase response of the sensor under varying tensile strain.
(b) Impedance variation as a function of tensile deformation.
(c) Inductance change under different tensile strain conditions.
(d) Voltage response under tensile deformation.
(e) Reflection coefficient (S11) characteristics under tensile strain.

**Table S1. Estimated effective relative permittivity and capacitance of the PDMS encapsulation layer under tensile deformation.**


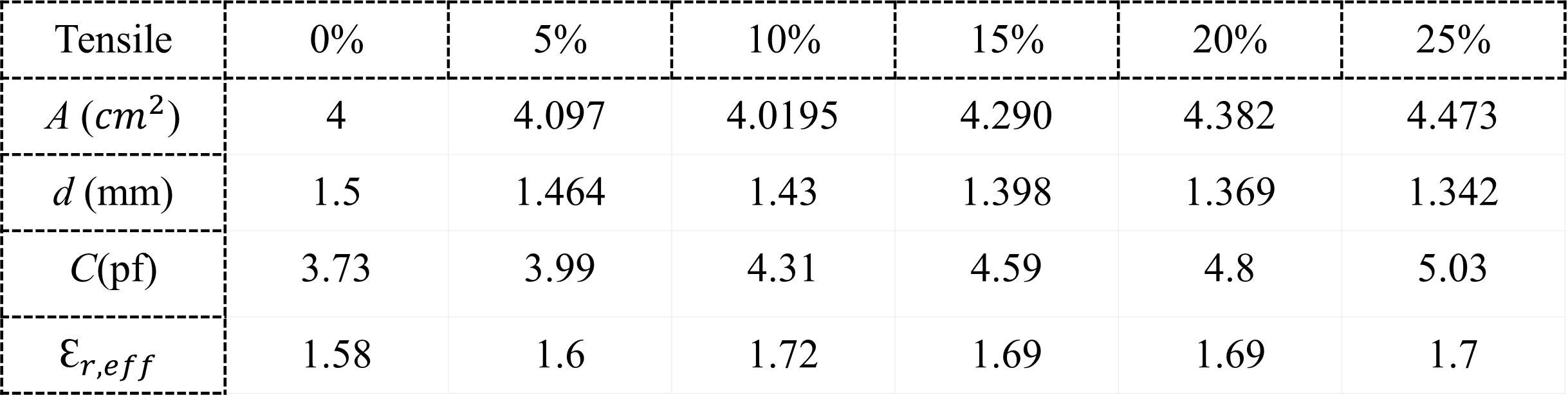

$$\boldsymbol{C=}\text{Ɛ}_{\boldsymbol{0}}\text{Ɛ}_{\boldsymbol{r}}\frac{\boldsymbol{A}}{\boldsymbol{d}}$$

$$\text{Ɛ}_{0}:\text{Vacuum permittivity (a physical constant, 8.854 × 10⁻¹² F/m)}$$

$$\text{Ɛ}_{r}:\text{Relative permittivity of the material}$$

*A* : Area

*d* : Separation distance

*C* : Capacitance
